# Schwannoma of the Tongue in a Paediatric Patient: A Case Report and 20-Year Review

**DOI:** 10.1155/2014/780762

**Published:** 2014-07-14

**Authors:** Nitin Bhola, Anendd Jadhav, Rajiv Borle, Gaurav Khemka, Umesh Bhutekar, Sanatan Kumar

**Affiliations:** Department of Oral and Maxillofacial Surgery, Sharad Pawar Dental College and Hospital, Wardha, Maharashtra 442004, India

## Abstract

Schwannomas (Neurilemmomas) are benign, encapsulated, slow-growing, and usually solitary tumours originating from Schwann cells of the peripheral nerve sheath with uncertain etiology. Approximately 25–48% of cases are seen in the head and neck region, of which 1% appears in the oral cavity. Lingual schwannoma can affect all age groups with peak incidence between the third and sixth decade. We report a rare case of lingual schwannoma in a 14-year-old girl complaining of asymptomatic swelling over lateral border of tongue since two years. Clinical examination revealed a nodule 1.5 × 1 cm in size, rubbery, nontender, smooth at right lateral border of tongue covered by normal mucosa, with no cervical lymphadenopathy. Excisional biopsy of the lesion was done under local anaesthesia. The histological sections spindle cells with thin wavy nuclei arranged as typical Antoni A (with Verocay bodies) and Antoni B areas. Nuclear palisading distribution (typical of a schwannoma) was readily identifiable. The patient was recurrence-free after one year.

## 1. Introduction

Schwannoma, also called neurilemmoma, is a solitary, benign, encapsulated, and slow growing tumor, arising from the neural sheath's Schwann cells of the peripheral, cranial (except for the optic and olfactory), spinal, and autonomic nerves [1]. Schwann cells form a thin outline around each extracranial nerve fiber and wrap larger fibers with an insulating membrane, myelin sheath, to enhance nerve conductance. As nerves exit the brain and spinal cord, there is a transition from myelination by oligodendrocytes to myelination by Schwann cells. Schwannomas arise when proliferating Schwann cells form a tumor mass encompassing motor and sensory peripheral nerves.

The cause of the tumor is unknown. However, some etiological factors are conjectured, such as spontaneous growth, external injury, chronic irritation, or exposure to radiation [2]. Approximately 25–48% arises in the head and neck region [3]. Schwannoma accounts for just over 1% of benign tumours reported in the oral cavity; they are the most commonly encountered nerve sheath tumours in this location [4]. Peripherally, the commonest location is the tongue [5], followed by the palate, floor of mouth, buccal mucosa, and mandible. Only 50% of these tumors have a direct relation with a nerve. In the tongue, the distinction between hypoglossal, glossopharyngeal, and lingual nerve origin is difficult, given their proximity. The peak incidence is between the third and sixth decades, with no predilection for gender or race [6].

The size and locations of lesions determine the presence and intensity of symptoms. The tumour is normally solitary, smooth-surfaced, slow growing, and generally asymptomatic. It may occasionally cause pain or discomfort. The goal of treatment is complete excision, which results in low rates of recurrence [7]. Malignant transformation is rare and incidence of malignant schwannomas ranges from 8% to 13.9% [8]. The following case is a rare case report of a lesion occurring in the tongue of a 14-year-old girl as the peak incidence of occurrence is between the third and sixth decade of life.

## 2. Case Report

**Figure 1:**
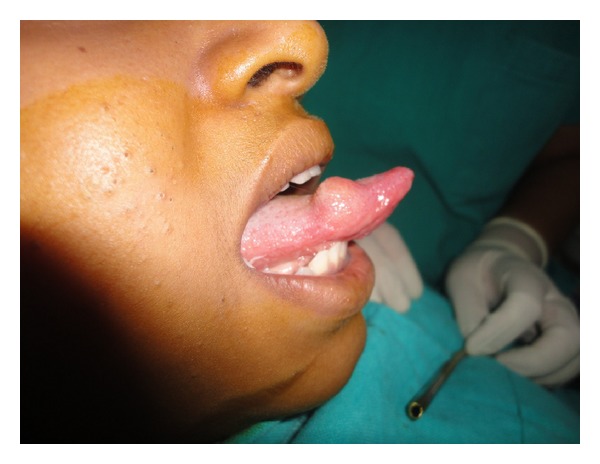
Asymptomatic nodule over right anterolateral side of tongue.

A 14-year-old girl presented to the department of oral and maxillofacial surgery for evaluation of a slow growing, occasionally painful nodule on right anterolateral side of tongue first noted 2 years back (Figure 1). Clinical examination revealed a nodule 1.5 × 1 cm in size, rubbery, nontender, and smooth at right lateral border of tongue covered by normal mucosa; no cervical lymphadenopathy was evident.

**Figure 2:**
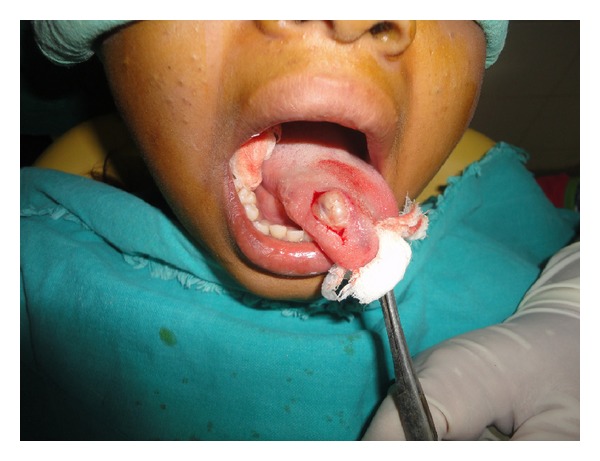
Encapsulated mass.

**Figure 3:**
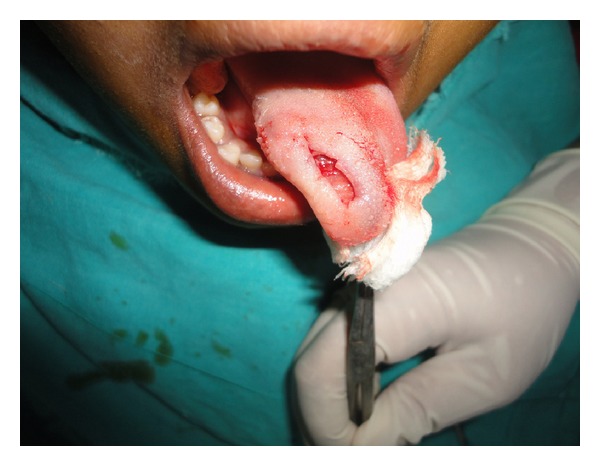
Complete excision.

**Figure 4:**
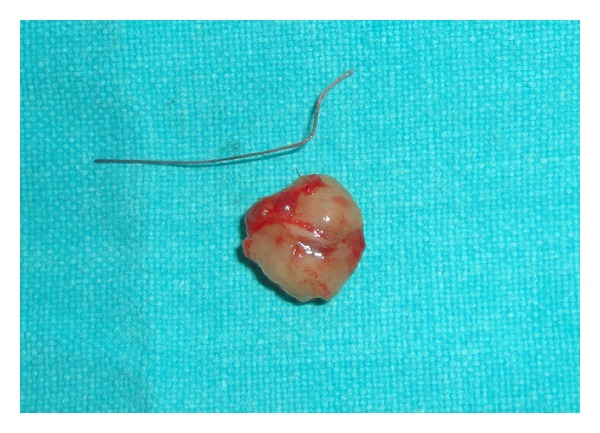
Excised mass.

Patient had no difficulty in chewing, swallowing, and phonation and there was no sensory or taste abnormalities by the patient. The remaining physical examination was unremarkable. The differential diagnosis included traumatic fibroma, neurofibroma, and benign lesions like tumors of salivary gland origin, leiomyoma, rhabdomyoma, lymphangioma, and hemangioma. Considering the size of the lesion an excisional biopsy was planned under local anesthesia. The mass was submucosal and once a mucosal flap was raised, the tumor was readily shelled out using blunt dissection (Figures 2, 3, and 4).

**Figure 5:**
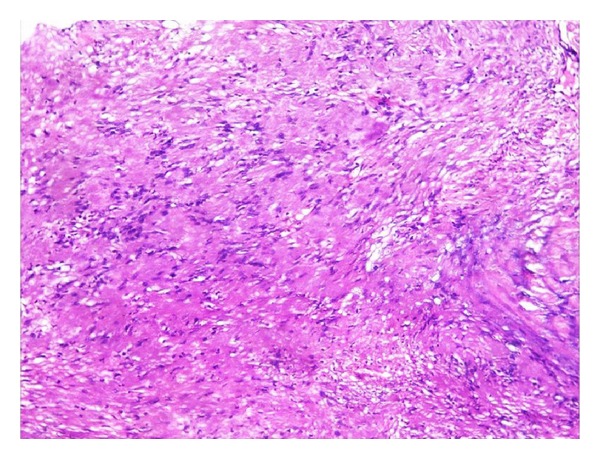
Spindle-shape neural cells arranged in Antoni A pattern with Verocay bodies.

The procedure and postoperative period were uneventful. Histopathological examination of the surgical specimen revealed a schwannoma, mainly composed of Antoni A pattern with Verocay bodies and Antoni B (Figure 5).

The patient has not shown any recurrence in follow-up period of 1 year.

## 3. Discussion

Almost a third of all schwannomas occurs in the head and neck region, but intraoral schwannomas are rare and account for 1% of the tumors growing in this region [4]. Because of their rarity, schwannomas are not generally part of the differential diagnosis of oral cavity lesions. Although the tongue is one of the most common tumour locations in the mouth, only 44 cases of lingual schwannoma have been reported in the English literature in the last 20 years [9]. However, on exploring PubMed we found 44 cases of lingual schwannoma reported in English literature in the past two decades (Table 1).

The mean age at diagnosis was 27 years. The age groups most affected were the 2nd (29.54%), 3rd (18.18%), and 4th (25%) decades of life. There was no gender predisposition. Two-thirds of tumours (i.e., 32) arose in the tongue with the remaining one-third (i.e., 12) affecting the tongue base. Standard treatment was transoral excision, performed in 93.18% of cases. However, for tumours located at the base of the tongue, in 2 cases CO_2_ laser was used, and in three other cases, the approach was transcervical (two submandibular and one transhyoid). All tumours in the tongue were treated with simple transoral excision. There were no reports of recurrence. There are only 13 cases reported in the literature categorized in the pediatric age group. The youngest of them were reported by Cinar et al. [10] and Enoz et al. [11], of 7-year-old, all being of male gender.

Lingual schwannoma can affect all age groups, being most commonly found between 30 and 40 years of age, without gender predisposition [6]. In this site, they usually appear as slow growing, progressive nodules, shown with symptoms that, when present, vary according to their size and location. The most common clinical presentation is a painless submucosal nodule with an average size of 2 cm [9]. Lingual schwannomas are rarely painful; they are usually noticed because of discomfort due to their position, such as difficulty in swallowing, chewing, and phonation [7].

Clinically, the schwannomas may be indistinguishable from other encapsulated benign tumors, so that biopsy and histological examination are essential to formulate a correct diagnosis. An excisional biopsy was performed in this patient, since treatment is exclusively surgical and usually enucleation of the mass is uncomplicated. In the present case, the patient did not present nerve injury after surgery, since the lesion was small and well defined. The option of complete resection was chosen on the basis of lesion form and size and to avoid recurrence.

The histological aspect of the lesion reported here was typical, consisting of a thin fibrous capsule and a tumor like proliferation formed by two types of tissue arrangements: Antoni type A and type B. The Antoni type A tissues are closely packed, forming bundles with elongated, palisaded nuclei. Free bands of amorphous substance between the rows of nuclei constitute the so called Verocay bodies which under electron microscope, appear to be composed of thin cytoplasmic processes with small amount of collagen and basal laminar material showing frequent redoubling, while in the Antoni B tissue it has less number of cells and less organization, where the fusiform cells are widely separated, dispersed in a loose and random fashion with a network of delicate reticulated fibers. Nuclear palisading distribution (typical of a schwannoma) was readily identifiable. The acid S-100 protein test was not performed, as the hematoxylin-eosin stained sections conclusively confirmed the diagnosis.

## 4. Conclusion

The schwannoma of the tongue is extremely rare, especially in children, and there are very few similar case reports in the present literature. Hereby we would like to add one new case of a schwannoma of the tongue as an example of a lesion which is often not taken into account during clinical practice or even considered as a possible diagnosis. Given the rarity of this lesion, a careful consideration is warranted as this may be clinically indistinguishable from fibroma, neurofibroma, and benign lesions like tumors of salivary gland origin, leiomyoma, rhabdomyoma, lymphangioma, and hemangioma. The definitive diagnosis requires a histopathological evaluation. Treatment is complete surgical excision of the lesion which does not result in any recurrence. The chance of malignant transformation of these tumors is unlikely.

## Figures and Tables

**Table 1:** Demographic characteristic of cases reported in past 20 years' literature.

Name of author(s) and year of publication	Age (years)/sex	Site of occurrence	Surgical approach	Follow-up
				(in months)
López and Ballestin, 1993 [12]	M/24	Tongue	Transoral	
	M/33	Tongue	Transoral	

de Bree et al., 2000 [13]	F/24	Base of tongue	Submandibular	

Pfeifle et al., 2001 [5]	M/18	Tongue	Transoral	
	F/30	Tongue	Transoral	

Mevio et al., 2003 [14]	F/35	Tongue	Tran oral	

Bassichis and McClay, 2004 [15]	M/9	Base of tongue	Transoral	60

Cinar et al., 2004 [10]	M/7	Tongue	Transoral	

Nakasato et al., 2005 [16]	F/9	Base of tongue	Transoral	17

Hwang et al., 2005 [2]	M/23	Tongue	Transoral	6

Vafiadis et al., 2005 [17]	M/18	Tongue	Transoral	36

Ying et al., 2006 [18]	F/26	Base of tongue	Transoral	

Hsu et al., 2006 [19]	M/20	Base of tongue	Transoral	3
	M/45	Tongue	Transoral	203
	F/12	Tongue	Transoral	13
	M/38	Tongue	Transoral	135
	F/15	Tongue	Transoral	136
	M/25	Tongue	Transoral	28
	F/32	Base of tongue	Transoral	63
	M/9	Tongue	Transoral	56
	F/39	Base of tongue	Transhyoid	23
	F/39	Tongue	Transoral	137

Mehrzad et al., 2006 [20]	M/49	Base of tongue	Transoral (CO_2_ LASER)	3

Enoz et al., 2006 [11]	M/7	Tongue	Transoral	60

Patnayak et al., 2007 [21]	F/45	Tongue	Transoral	

Ballesteros et al., 2007 [22]	F/31	Base of tongue	Transoral (CO_2_ LASER)	

Batra et al., 2007 [23]	M/33	Base of tongue	Transoral	6
	M/33	Base of tongue	Transoral	9

Sawhney et al., 2008 [6]	F/37	Base of tongue	Submandibular	

Ferreti Bonan et al., 2008 [24]	F/46	Tongue	Transoral	12

Pereira et al., 2008 [25]	M/12	Tongue	Transoral	12

Cohen and Wang, 2009 [7]	M/77	Tongue	Transoral	
	F/19	Tongue	Transoral	

Gupta et al., 2009 [26]	F/18	Tongue	Transoral	

Karaca et al., 2010 [27]	F/13	Tongue	Transoral	12

Naidu and Sinha 2010 [28]	M/12	Tongue	Transoral	3

Lukšić et al., 2011 [29]	M/10	Tongue	Transoral	60

Nisa et al., 2011 [30]	F/38	Tongue	Transoral	

Husain et al., 2011 [31]	F/10	Tongue	Transoral	12

Jeffcoat et al., 2010 [32]	M/68	Tongue	Transoral	

Catalfamo et al., 2011 [33]	M/28	Tongue	Transoral	

Manna et al., 2012 [34]	M/15	Tongue	Transoral	

Lira et al., 2013 [35]	F/28	Tongue	Transoral	

Moreno-García et al., 2014 [36]	F/13	Tongue	Transoral	12
